# Supplementary material for: Qualitative study of in-kind incentives to improve healthcare quality in Belize: Is quality work better than wealth?
Source: PLoS One. 2023 Aug 18;18(8):e0290457. doi: 10.1371/journal.pone.0290457 (PMC10437859; doi:10.1371/journal.pone.0290457)
Supplement: Attachment — Submitted filename: Belize QIF Incentives and team motivation Resp.Mar.15.23.docx [file pone.0290457.s003.docx]

| Reviewer Comments | Response from Authors |
| --- | --- |
| 1. Is the manuscript technically sound, and do the data support the conclusions?  The manuscript must describe a technically sound piece of scientific research with data that supports the conclusions. Experiments must have been conducted rigorously, with appropriate controls, replication, and sample sizes. The conclusions must be drawn appropriately based on the data presented.  Reviewer #1: Yes  Reviewer #2: Partly | We express our gratitude to both reviewers for their constructive comments. We have taken their feedback into consideration and made appropriate revisions, with the aim of enhancing the overall quality of our manuscript. |
| 2. Has the statistical analysis been performed appropriately and rigorously?  Reviewer #1: N/A  Reviewer #2: No | Given that this was a qualitative study, we did not conduct statistical analyses. |
| 3. Have the authors made all data underlying the findings in their manuscript fully available?  The [PLOS Data policy](http://www.plosone.org/static/policies.action#sharing) requires authors to make all data underlying the findings described in their manuscript fully available without restriction, with rare exception (please refer to the Data Availability Statement in the manuscript PDF file). The data should be provided as part of the manuscript or its supporting information, or deposited to a public repository. For example, in addition to summary statistics, the data points behind means, medians and variance measures should be available. If there are restrictions on publicly sharing data—e.g. participant privacy or use of data from a third party—those must be specified.  Reviewer #1: No  Reviewer #2: Yes | We have now included the codified dataset as supplementary materials. However, we cannot share the deidentified transcripts due to the potential compromise of informant confidentiality. |
| 4. Is the manuscript presented in an intelligible fashion and written in standard English?  PLOS ONE does not copyedit accepted manuscripts, so the language in submitted articles must be clear, correct, and unambiguous. Any typographical or grammatical errors should be corrected at revision, so please note any specific errors here.  Reviewer #1: Yes  Reviewer #2: Yes | Thank you, we reviewed the manuscript again to ensure proper use of the English language. |
| Reviewer #1: There are a number of items from the COREQ guidelines which have not been included in the manuscript in it's current form. These should be included in the revision. | Thank you for taking the time to read our manuscript and provide constructive comments. We have revisited the COREQ guidelines and made an effort to provide more precise descriptions. |
| 1. On lines 21-23 of the background, the authors should not reveal the results of the study by indicating that improvements were observed but instead they should specify what the gap in the literature is or the problem statement as well as the aim of the study. | We modified the abstract to include the knowledge gap and aim of the study.  Background: There is a sparsity of knowledge of the specific mechanisms through which financial and non-financial incentives impact the performance of health teams. This study aims to address this knowledge gap by examining an in-kind incentives program for healthcare teams implemented in three districts in Belize (2012―2022) as part of the Salud Mesoamerica Initiative, which aimed to improve healthcare quality *Lines 20-24 (clean version)* |
| 2. Line 24, 25 and 30. Please avoid using the passive voice “was performed”, “were conducted” and “were identified”. Instead use the active voice by starting the sentence with the words “we performed”, “we conducted” and “we identified”. | We revised the abstract and manuscript to use active voice. |
| 3. Line 37 of the conclusion. The authors need to make clear which activities they are referring to here as this paragraph is about in-kind incentives. | Thank you for pointing this out. We have edited the Conclusion to make it clearer:  Rather than focusing on tangible incentives, explicitly incorporating standardized measurements and supportive supervision in the routine work of the Ministry of Health and Wellness could have longer lasting effect than quality improvement.  *Lines 20-24 (clean version)* |
| 4. Line 48 – please correct typing error. Should read as “middle-income”. | Corrected.  *Line 51 (clean version)* |
| 5. Line 113 – please write all the initials of the author ABC as it is not clear if you are referring to AEBC. | We changed the initials to AEBC throughout the manuscript. |
| 6. The authors should make it clear whether or not they established a relationship with the participants prior to study commencement. They also need to specify the interviewers characteristics such as any bias, assumptions, reasons and interests in the research topic. This is one of the requirements needed to publish qualitative research according to the COREQ guidelines. | We clarified that a relationship was not established before the study as follows:  AEBC had no prior knowledge of the participants prior to the study, and only interacted with them in advance for the purpose of scheduling the interviews.  *Lines 158-159 (clean version)*  We also added additional information to describe with more detail the interviewers characteristics:  AEBC (MD, male, graduate student) conducted all interviews through audio conferences using Zoom or by telephone. […] AEBC had no financial or professional interest in the QIF, as he was not involved in its design or implementation and had no prior experience with incentive programs. His primary motivation was to gain a deeper understanding of hospital efficiency and healthcare quality. The findings of the study and the potential continuation or discontinuation of the QIF did not result in any personal benefit or employment opportunities for any of the researchers involved in this study.  *Lines 141-149 (clean version)* |
| 7. Line 121 – please clarify if the two mock interviews were conducted as part of the pilot testing of the questionnaire. | We added the following clarification in the text:  AEBC received a 2-hour training on interviewing techniques and conducted two mock interviews complement training and pilot the instruments.  *Lines 143-144 (clean version)* |
| 8. As part of the data collection section, please specify if repeat interviews were carried out and if so, how many. | No repeat interviews were carried out. We added the following clarification in the text:  We did not conduct repeat interviews and did not request feedback from participants. *Lines 161-162 (clean version)* |
| 9. Also in the data collection section, please include if field notes were made or not. | No repeat interviews were carried out. We added the following clarification in the text:  AEBC took notes during interviews, which assisted transcribing.  *Lines 161 (version with changes)* |
| 10. In addition, authors should include if transcripts were returned to the participants for comment or correction. | We added the following clarification in the text:  We did not conduct repeat interviews and did not request feedback from participants. *Lines 161-162 (clean version)* |
| 11. The authors should explain how they assessed data saturation. | We included the following text:  Our criterion for saturation was hearing repeated themes during interviews.  *Lines 134-135 (clean version)* |
| 12. In the analysis section, the authors should specify which software (if any) was used for the analysis. If manual coding was performed, this should be stated. | We included the following clarification:  The researchers manually coded using open coding to identify patterns and themes based on the data.  *Lines 167-168 (clean version)* |
| 13. The authors need to state whether or not the participants provided feedback on the findings. | We added the following clarification in the text:  We did not conduct repeat interviews and did not request feedback from participants |
| 14. Line 144 – 145. The word “the” is missing between “of” and “informants” in the sentence that reads: “Nevertheless, two of informants from hospitals…” | Corrected. |
| 15. Line 150-151. The authors state that under the three tropics, 11 major themes and 27 sub-themes were identified. More clarity should be provided as this statement seems to imply that deductive coding was used yet the authors had previously mentioned that they used open coding (line 134). The authors should make it clear whether they simply used the topic headings to classify themes found through open coding or it they used the topic headings to find themes and patterns. | Topic headings were used to organize themes and subthemes after coding was completed. We added the following clarification:  After completing coding, we organized themes and subthemes identified through open coding under three topics *Lines 181-183 (clean version)* |
| 16. Lines 245-246 need to be deleted as they are repeated on lines 247-248 | Eliminated repeated text. |
| 17. Line 246. The word “were” in the phrase “criteria were used” seems out of place and should be omitted. | Rephrase the sentence as follows:  Moreover, it was not always clear how the criteria were utilized to calculate indicators.  *Line 297 (clean version)* |
| Reviewer #2: The authors report to valuable work in understanding the elements which contributed to the quality improvement within the QIF supported programs. The manuscript is very well written and is is grammatical correct. | Thank you for your feedback. We have made further improvements to our editing and have addressed the issues you raised. |
| Major reasons for rejection: The methodology followed is not to standard and this is the main reason for the rejection, which does not comply with the Qualitative study reporting guidelines required by PLoS One. In particular, there is no that authors used the Consolidated criteria for reporting qualitative research (COREQ) checklist or Standards for reporting qualitative research (SRQR) checklist. If this was used, further detail is required and deviations to this checklist noted. | In line with your suggestion, and the comments of the other reviewer, we have added more details to comply with the COREQ and SRQR checklists. |
| Within the Data Collection section, while there is mention of the IRB approval, no indication if consent was requested of Key Informants, what was contained in the consent process and how was this conducted. Further no indication if participants were reimbursed for their time, and if not this ought to be mentioned. | Thank you for pointing this out. We have added the following clarifications:  Informed consent was obtained orally before each interview from all participants. […] Participants could stop the interview at any time and did not receive compensation. […] The study was reviewed and approved by the Institutional Review Boards at Cornell University (Ithaca, NY) (Protocol ID#: 2103010245) and at Belize’s MOHW (Ref. GEN/147/01/21(12) Vol. V).  *Lines 157-164 (clean version)* |
| Minor comments: 1. The program was conducted between 2013 and 2017, while the key informant interviews were conducted between April-June 2021. There is no indication for the reasons for the lengthy period between program ending and the data collection. This could be a limitation in recall bias and should be acknowledged in the limitations section. | Thank you. We have clarified that some components (measurements) continued, although the latest incentive before our study was in 2017. We have included recall bias as a limitation as follows:  Third, we conducted our study almost four years after the last incentive was given, which could have created recall bias and the omission of important details.  *Lines 384-385 (clean version)* |
| 2. The reason in-depth interviews with staff working within the program was not considered? I believe some of the limitations noted of the study could have been resolved by their inclusion. Further this would have prevented the mainly high level view of the incentives provided. | As stated in our manuscript, all informants worked within the program. The implementation of the program in Belize was carried out by making functional adjustments within the Ministry of Health and Wellness, rather than contracting separate staff. See:  The majority of informants were medical doctors or nurses at hospitals, with one informant at regional-level manager and another working in a rural clinic. However, two of the informants from hospitals were nurse managers of several rural clinics.  *Lines 177-178 (clean version)* |
| 3. A description of the interview guide was provided. More adequate would have been to provide the actual guide in the appendices of the manuscript submission. | Thank you for this suggestion. We have now included the interview guide in the Annex B. |
| 4. A description of the data analysis is given, however no indication if this was manually done or with the aid of software tool. | We included the following clarification:  The researchers manually coded using open coding to identify patterns and themes based on the data. *Lines 167-168 (clean version)* |
| 5. While Table 1 provides the themes which emerged from interviews, there is little indication of how these themes related to one another to one another and/or if these themes coincide with any particular theoretical framework | We derived the themes through the open coding process and did not rely on any specific framework. To make them more accessible, we categorized them into three topics. We added the following clarification in the text:  After completing coding, we organized themes and subthemes identified through open coding under three topics *Lines 181-183 (clean version)* |
| 6. the definition of intrinsic and extrinsic motivation factors is poorly defined. Since this is a major distinction and has underlying differentiation to the overall conclusion in the study, a better description with examples within the program could be indicated. | Thank you for your suggestion. We have expanded the description of intrinsic and extrinsic motivation in our manuscript:  Extrinsic motivation, which stems from external factors such as financial rewards and verbal recognition, can be enhanced by these incentives [5]. However, research suggest that such incentives may lead to unintended long-term consequences [5]. They can create a transactional approach to performance and undermine intrinsic motivation [6,7], which is characterized by the drive to engage in a behavior for its own sake and because it is meaningful, such as having empathy for patients [5].  *Lines 60-65 (clean version)* |
| 7. Lines 315-319, present a limitation of the study and is related to point 2 above where the inclusion of staff may have added value to gaining the views of both staff and management teams. | We agree that a larger sample may have added a more detailed perspective. However, as we describe in our manuscript, the sample does include staff with management and health service provision perspectives. Note that in Belize, due to staffing limitations, many people take multiple roles. See:  The majority of informants were medical doctors or nurses at hospitals, with one informant at regional-level manager and another working in a rural clinic. However, two of the informants from hospitals were nurse managers of several rural clinics.  *Lines 181-183 (clean version)* |
| 8. With a bit more description in the results section, figure 1 may not be needed. | Thank you for your valuable suggestion! We appreciate your input. We would like to highlight that Figure 1 is a helpful addition to the manuscript as it presents a concise summary of the results, which is also recommended in the COREQ checklist. We believe that Figure 1 will be useful for readers who are looking for a quick overview of the research findings. |
