# Supplementary material for: Qualitative study of in-kind incentives to improve healthcare quality in Belize: Is quality work better than wealth?
Source: PLoS One. 2023 Aug 18;18(8):e0290457. doi: 10.1371/journal.pone.0290457 (PMC10437859; doi:10.1371/journal.pone.0290457)
Supplement: Attachment — Submitted filename: Belize QIF Incentives and team motivation Resp.Jun.16.23.docx [file pone.0290457.s004.docx]

| Comments | Response from Authors |
| --- | --- |
| **Editorial Comments** | Thank you for your valuable suggestions. We have incorporated important revisions into our manuscript, aiming to provide a more comprehensive and robust perspective on our results. |
| Thank you for your revisions in response to the comments. Please respond carefully to the reviewers comments that request you to report in accordance with the COREQ guidelines. Perhaps a checklist can be appended in response to the comment. | As suggested by you and one of the reviewers, we have appended the COREQ checklist to our manuscript. See supplementary materials. |
| I would also recommend having a qualitative method expert advise you on the analysis that is presented. The coding process needs to be described in more detail and the use of a framework would make the analytic process more robust. | Thank you. We expanded the description of the coding process and included details of the reference framework used, as follows:  “AEBC and DRZ conducted a collaborative analysis of the data using qualitative content analysis. We systematically categorized the transcribed data to classify, summarize, and tabulate it. We employed an open coding approach, manually assigning codes to the data, enabling us to identify patterns and themes in an inductive manner, without relying on any preexisting framework. To perform the analysis, we employed a methodology akin to the one suggested in the existing literature [15], utilizing Spreadsheet Software (MS Excel 365, Seattle, Microsoft) for qualitative content analysis. We recorded quotes from interviews in the spreadsheet and assigned each to a separate row. All responses were analyzed collectively, without considering the specific questions that were answered. In a neighboring column, the researchers provided a descriptive preliminary code per quote. Through an iterative process, similar codes were merged until no further merging was possible.” (page 9, lines 169-178) |
| Recall bias and risk of bias are not meaningful constructs in the qualitative approach. In fact in keeping with the approach please comment on data saturation, reflexivity and truth value of the research. Table 1 is also not in keeping with the qualitative approach and is not required instead a description of the participants is needed. | We removed recall bias, risk of bias from the manuscript, and replaced Table 1 with a description of participants.  We added the following text in the discussion to comment on data saturation: “Although a larger sample could have provided us a richer perspective, the degree of agreement between respondents already reveals the most salient features of the QIF. In the end, we interviewed over one-fourth of all potential informants.” (Page 20, Lines 417-419)  To comment on reflexivity and truth value, we included the following:  “It is worth noting that our research team consisted of individuals from diverse backgrounds, providing a range of views that enhanced our analysis and offered a broader perspective.” (page 20, lines 425-427)  We also believe that the COREQ checklist, and data provided in the Supplementary files increase the credibility, dependability and auditability of our study. |
| The manuscript would benefit from English language editing as pointed out by reviewer 1. | We have closely reviewed the manuscript for English language use. |
| Please also reference in the discussion the debate regarding pay for performance ..the jury is still hung and the evidence of benefit is not convincing. We look forward to a revision that considers these issues highlighted | Thank you, this is indeed an important highlight. We included the following paragraph in the discussion:  “In the context of health incentive programs, our study makes several important contributions. Firstly, it clarifies how in-kind incentives effectively enhance performance by revealing their operational mechanisms. Criticisms have been raised against incentive schemes for their excessive emphasis on short-term outcomes, neglecting their systemic effects [20]. Therefore, it is crucial to emphasize the effective implementation of incentive schemes, as the process itself plays a significant role [20]. Secondly, our study highlights the key design elements of the incentive program that were crucial in achieving positive results. In low- and middle-income countries (LMICs), there is still a lack of understanding regarding the specific mechanisms through which incentive schemes influence health worker performance [19]. Thirdly, our study introduces a new layer of discussion by exploring the potential of non-financial incentives in improving intrinsic motivation. Although there is a growing body of evidence demonstrating the positive impact of incentives on healthcare quality [17, 18], most studies primarily compare incentive schemes with the existing practices or other financing methods [17]. Only a few studies have focused on interventions that incorporate the main components of incentive programs, such as routine measurements, data-sharing systems, and supervision, without providing financial incentives.” (page 18, lines 365-378) |
| **Reviewer 1** | Thank you for you time reviewing our manuscript. We appreciate your constructive comments. |
| 1. Line 41 (clean version): Please confirm if the word “than” is a typo in the statement “…explicitly incorporating standardized measurements and supportive supervision in the routine work of the Ministry of Health and Wellness could have longer lasting effect than quality improvement.” I’m not clear if the authors meant to say “on”. | Thank you for catching this typo. We have corrected the abstract and checked the entire manuscript (see page 3, line 43). |
| 2. Please check grammar on line 144 (clean version) that reads “…conducted two mock interviews complement training and pilot the instruments.” I’m not sure if the authors meant to say “…two mock interviews to complement training…”? | We rephrase the sentence as follows:  “AEBC received a 2-hour training on interviewing techniques and conducted two mock interviews to complement training and to pilot the instruments.” (see page 8, lines 143-144) |
| **Reviewer 2** | Thank you for your time reviewing our manuscript. Your suggestion helped us include a couple of items that we had previously missed. |
| The authors have taken the time to respond to each of the queries raised and have paid attention to some of the fundamental concerns raised in the previous review. Thank you. I am though at this stage not able to fully confirm that the COREQ guidelines have been followed to the 32 point scale. I would ask that the COREQ 32 point checklist is included in a revised submission, allowing to compare the text included to the checklist, and assessing if analysis was conducted to standard. Further, I am assuming the COREQ standard is applied as this was not indicated in methods along with the appropriate reference. If the Standards for reporting qualitative research (SRQR) checklist was used, this should likewise be included as an appendix and noted in text to indicate the standard applied. | Thank you. We have now included the following text in our manuscript and attached the COREQ checklist as a supplementary file:  “We followed the Consolidated criteria for reporting qualitative research [17] to present our results.” (page 10, line 188) |
